# Experience-dependent reactivations of ventral tegmental area neurons in the rat

**DOI:** 10.1186/1471-2202-12-S1-P107

**Published:** 2011-07-18

**Authors:** José L Valdés, Bruce McNaughton, Jean-Marc Fellous

**Affiliations:** 1 Department of Physiology and Biophysics, I.C.B.M., Faculty of Medicine, University of Chile, Chile; 2 Department of Neuroscience, Canadian Centre for Behavioural Neuroscience, The University of Lethbridge, CA, USA; 3 Department of Psychology and program in Applied Mathematics, University of Arizona, USA

## 

The hippocampus stores information during the acquisition of new memory episodes. These memories are replayed during sleep as part of a memory consolidation process. The neural mechanisms underlying these reactivations are currently under investigation. One hypothesis is that reactivation occurs as a result of local attractor dynamics within the structure in which they occur. Another possibility is that reactivation in these various areas is at least in part inherited from one or several other structures that project to them. Theoretical and experimental work on reinforcement learning have proposed many ways in which learning can be modulated by the value associated with a stimulus. Beyond initial memory acquisition, however, it is still unclear why specific memory items are consolidated and others are not [1,2]. One possibility is that, as with memory acquisition, the consolidation process is modulated by the value associated with a specific memory item. Research has shown that this value may be at least in part encoded by subcortical structures such as the ventral tegmental area (VTA) [3,4].

We provide new evidence in the rodent that 45% VTA neurons are sensitive to and selective for different types of stimuli. In three different tasks involving various amounts spatial and reward components, we show that putative dopaminergic VTA neurons strongly reactivate during a rest period following the tasks. This reactivation takes the form of population-wide activity patterns lasting from a few 100 ms up to a few seconds. In the non-spatial task, a statistical analysis of this reactivation using the explained variance measure showed that most of the reactivation relies on the activity of stimulus-sensitive neurons. Stimulus insensitive neurons exhibited significant reactivation if the task used involved motor and spatial components.

VTA has widespread connections to the rest of the brain, including hippocampus and neocortex. Almost all experiments that have shown memory trace reactivation were based on tasks that involved rewards. The selective reactivation of non-GABAergic cells in the VTA suggests that reactivations in hippocampus and cortex could be modulated by the VTA. The finding that reward-sensitive neurons primarily reactivated when rewards were present during the task suggests a mechanism by which hippocampal and neocortical reactivations can be modulated by the value of the memory items they encode. In turn, cortex and hippocampus could modulate VTA activity as part of a loop in which memory content and memory value are dynamically and selectively established. Future conceptual and computational models of reinforcement learning and memory consolidation could account for the type of selectivity and dynamics of VTA neural firing we described here.
